# Visual record of intertidal disturbance caused by drift ice in the spring on the Atlantic coast of Nova Scotia

**DOI:** 10.12688/f1000research.4146.1

**Published:** 2014-05-16

**Authors:** Willy Petzold, Maike T. Willers, Ricardo A. Scrosati

**Affiliations:** 1 Department of Biology, St. Francis Xavier University, Antigonish, Nova Scotia, B2G 2W5, Canada

## Abstract

In the early spring of 2014, an unusually large amount of sea ice drifted from the Gulf of St. Lawrence, where it had been produced, towards the open Atlantic Ocean through the Cabot Strait, between Nova Scotia and Newfoundland, Canada. In early April, significant amounts of drift ice reached the Atlantic coast of mainland Nova Scotia. The ice floes persisted in those coastal waters for up to 16 days, depending on the location. During that time, the ice fragments caused extensive physical disturbance in rocky intertidal communities, removing high quantities of seaweeds and invertebrates. For example, at a location where the ice stayed for 9 days, the loss of macroalgal and invertebrate biomass was almost total. At a location where the ice stayed for 4 days, losses were lower, albeit still high overall. Such a magnitude of disturbance is not common on this coast, as sea ice had not reached the surveyed locations in the previous 4–5 years. We suggest that the frequency of ice scour events may help to predict intertidal community structure. This notion could be tested through multiannual surveys of ice conditions and biological communities along the Atlantic coast of Nova Scotia.

## Observation

The NW Atlantic coast exhibits cold-temperate conditions. As with similar systems in other parts of the world, the distribution and abundance of rocky intertidal species are greatly influenced by latitudinal changes in temperature and pelagic food supply ^1– 3^. Unlike most other temperate coastal systems, however, on the NW Atlantic coast, sea ice may affect considerably the survival of intertidal species and, consequently, the structure of biological communities.

**Figure 1.**
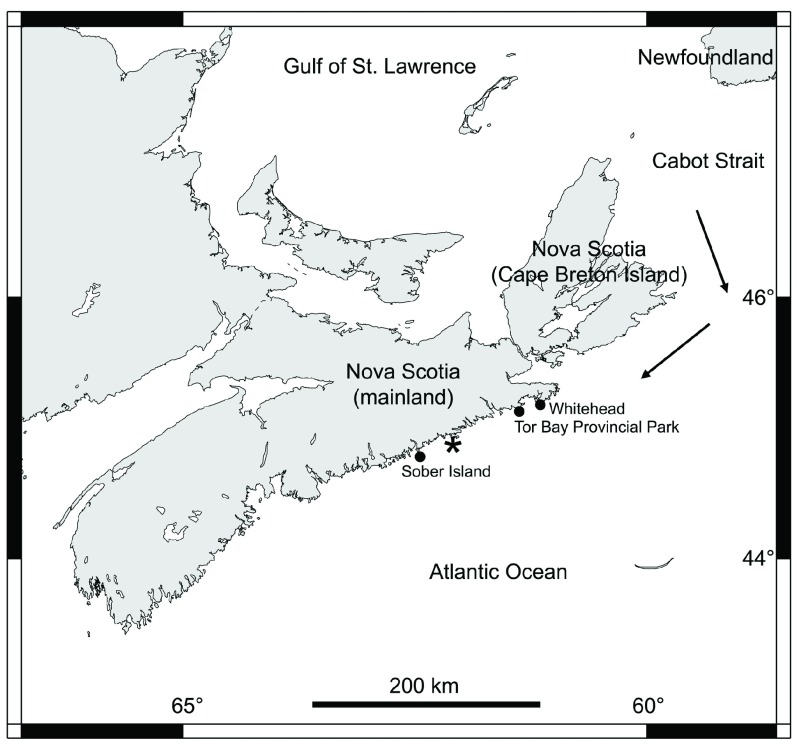
Map of Nova Scotia. The coastal locations from mainland Nova Scotia referred to in the text are indicated with black dots. The arrows indicate the direction that the sea ice originated in the Gulf of St. Lawrence normally follows when drifting out of the gulf. The asterisk shows the southernmost reach of the drift ice on the coast of mainland Nova Scotia in 2014, according to the Canadian Ice Service.

While a stable ice coverage of intertidal habitats (the ice foot) prevents benthic organisms from experiencing very low temperatures during low tides ^4^, the movement of ice fragments because of tides, currents, winds, and waves can severely damage or remove intertidal organisms ^5, 6^. On many NW Atlantic shores from relatively enclosed bodies of water, such as gulfs or bays, sea ice readily develops on the sea surface every winter, causing a great deal of disturbance in rocky intertidal communities when ice fragments move around ^7^. On the open Atlantic coast, however, ice does not form on the sea surface. Nonetheless, drift ice produced in enclosed bodies of water may still reach the open coast and cause damage there. Such is the case of the open Atlantic coast of Nova Scotia. Between mid-winter and early spring, sea ice produced in the large Gulf of St. Lawrence often drifts towards the Atlantic Ocean through the Cabot Strait, between Nova Scotia and Newfoundland ( Figure 1). The floating ice fragments then move southwards along the Atlantic coast. The extent to which the ice floes travel south varies between years, often being limited but reaching the central coast of mainland Nova Scotia in unusually extreme years ^8^ ( Canadian Ice Service).

**Figure 2.**
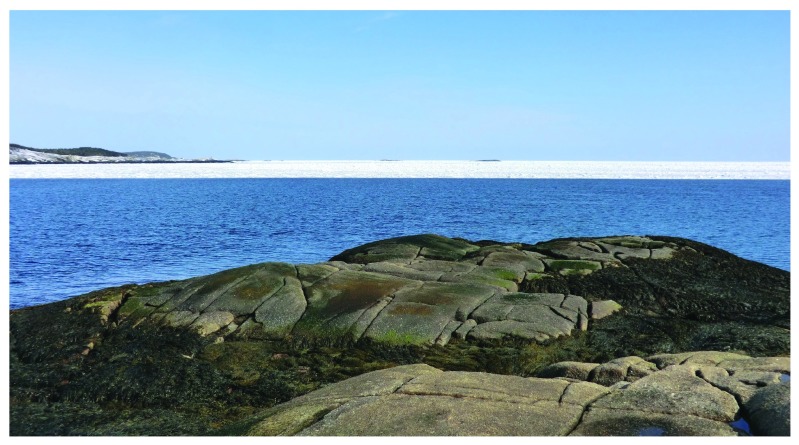
Whitehead just before the arrival of the drift ice. Picture taken at low tide in the afternoon of 3 April 2014 at a wave-exposed site in Whitehead, showing a full coverage of the intertidal zone by seaweed canopies and the drift ice approaching the shore. The sea surface was calm on that day.

**Figure 3.**
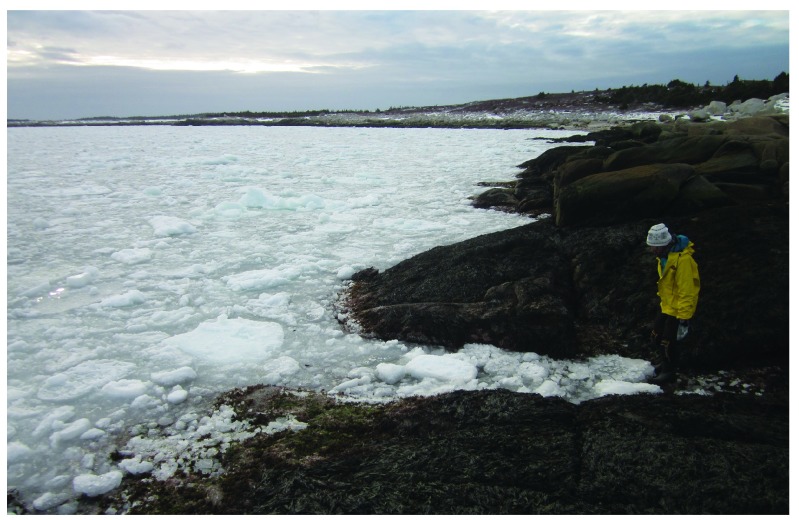
Whitehead at the time of arrival of the drift ice. Picture taken at low tide in the late afternoon of 3 April 2014 from the wave-exposed site in Whitehead shown in Figure 2. This picture shows the variable size of the ice fragments at the time of their first contact with the shore.

**Figure 4.**
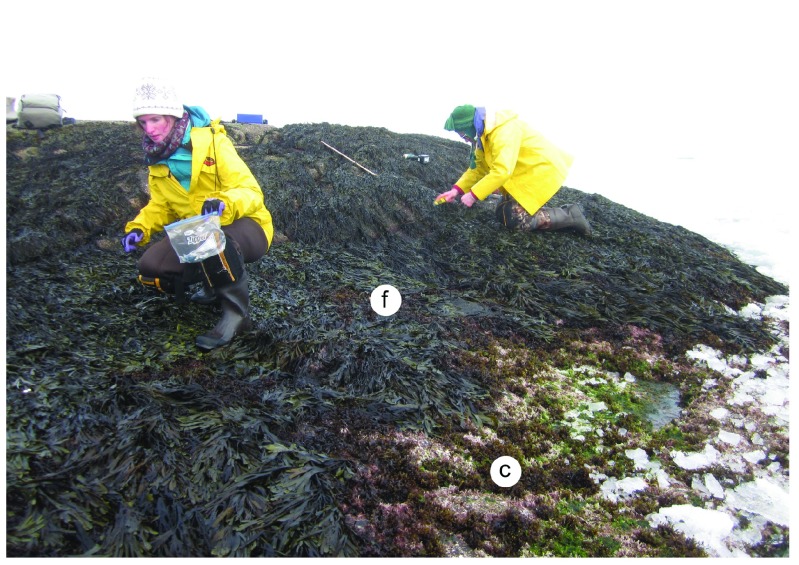
Whitehead at the time of arrival of the drift ice. Picture taken at low tide on 3 April 2014 at the wave-exposed site from Whitehead shown in Figure 2. This picture shows the intertidal zone covered by a *Fucus* canopy at high and middle elevations (f) and by *Chondrus crispus* and coralline algae at low elevations (c), which also exhibit the first ice fragments that contacted the shore on that day.

**Figure 5.**
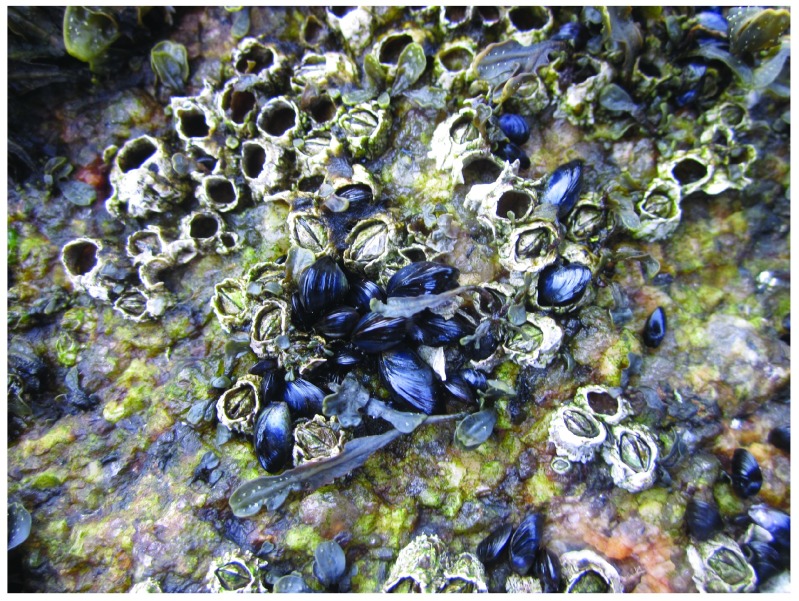
Whitehead at the time of arrival of the drift ice. Picture taken at low tide on 3 April 2014 at the wave-exposed site from Whitehead shown in Figure 2. This picture shows the mussels and barnacles that were abundant in understory habitats below the *Fucus* canopy, which was removed to take the picture.

**Figure 6.**
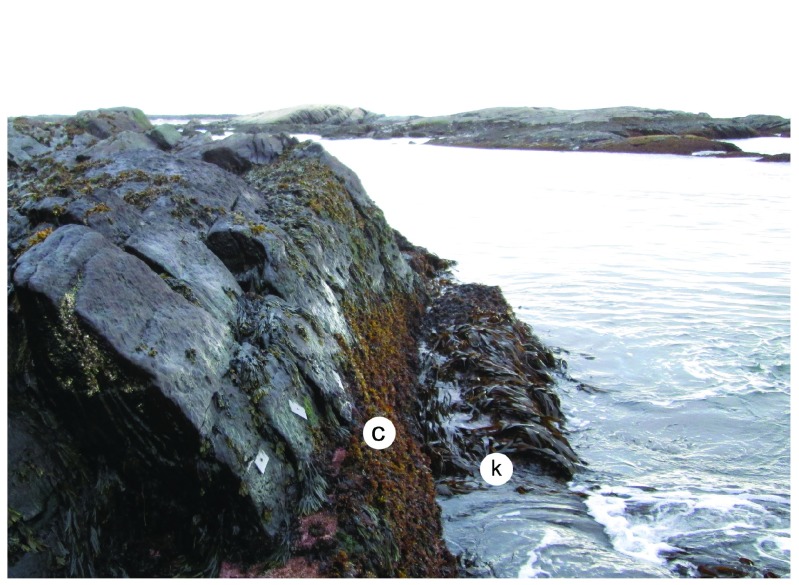
Tor Bay Provincial Park shortly before the arrival of the drift ice. Picture taken at low tide on 4 April 2014 at a wave-exposed site in Tor Bay Provincial Park, showing a well developed canopy of *Chondrus crispus* at middle-to-low elevations (c) and a kelp canopy at the lowest elevations (k). The little plates that are visible above the *C. crispus* zone were drilled into the rocky substrate to study barnacle recruitment. The sea surface was calm on that day, and sea ice was visible towards the horizon.

In 2014, a large amount of floating ice fragments came out of the Gulf of St. Lawrence between late winter and early spring. In its travel south along the Atlantic coast, the ice came in contact with an approximately 92-km-long stretch of coastline in mainland Nova Scotia ( Figure 1). Ice fragments varied widely in size, but together formed a relatively compact coverage of the sea surface ( Figure 2– Figure 3). Such a high influx of sea ice eventually devastated rocky intertidal communities. Before the arrival of the ice in early April, intertidal habitats were abundantly covered with seaweeds and invertebrates. For example, in Whitehead (45° 12' 43.5" N, 61° 10' 25.6" W, Figure 1), high and middle intertidal elevations from wave-exposed habitats exhibited a well developed canopy of *Fucus* algae ( Figure 4) and an abundance of mussels ( *Mytilus*) and barnacles ( *Semibalanus balanoides*) in understory habitats ( Figure 5). At middle and low elevations from wave-exposed habitats in Tor Bay Provincial Park (45° 10' 57.6" N, 61° 21' 19.4" W, Figure 1), a dense canopy of *Chondrus crispus* (a red alga) dominated the landscape, while, at the lowest intertidal elevations, kelp (mostly *Laminaria* and *Saccharina*) formed a conspicuous canopy that covered smaller algae, such as *C. crispus* and coralline algae, and a diversity of small invertebrates ( Figure 6).

**Figure 7.**
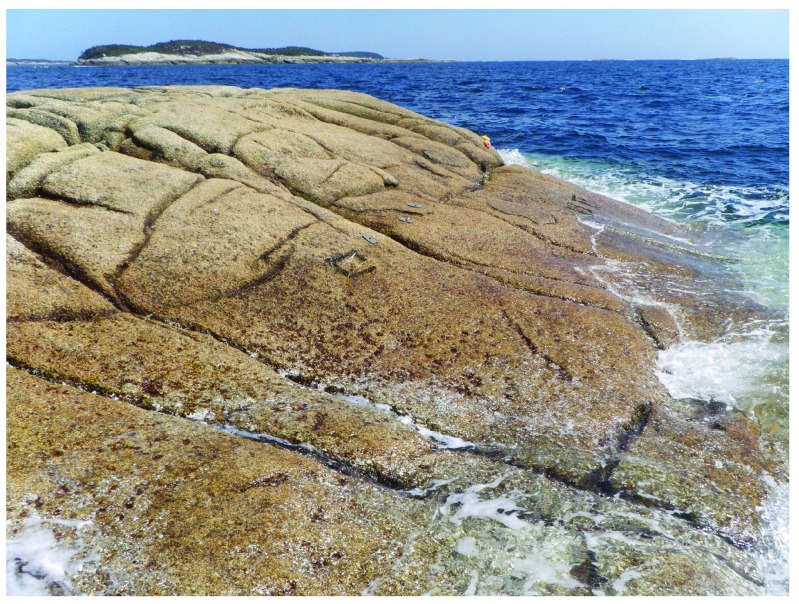
Whitehead after ice scour. Picture taken at low tide on 30 April 2014 at the wave-exposed site from Whitehead shown in Figure 4, showing the extreme removal of algae and invertebrates by the sea ice, which stayed for 9 days on the shore. The little barnacle recruitment plates visible in this picture were drilled to the rocky substrate at an elevation of approximately 2/3 of the full intertidal range (between chart datum, or 0 m in elevation, and the elevation where the barnacles located highest on the shore occurred before the ice scour).

**Figure 8.**
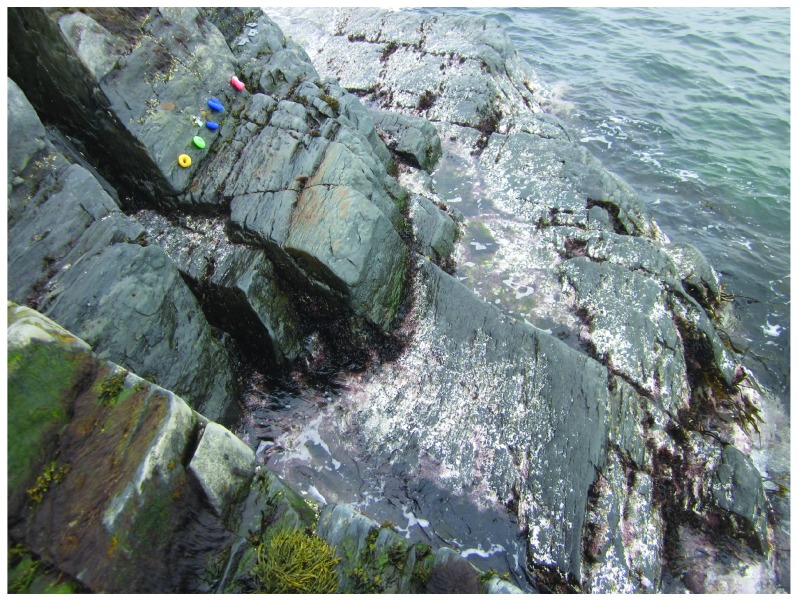
Tor Bay Provincial Park after ice scour. Picture taken at low tide on 27 April 2014 at the wave-exposed site from Tor Bay Provincial Park shown in Figure 6. This picture shows the almost complete loss of the macroalgal cover shown in Figure 6 because of the effects of ice scour.

**Figure 9.**
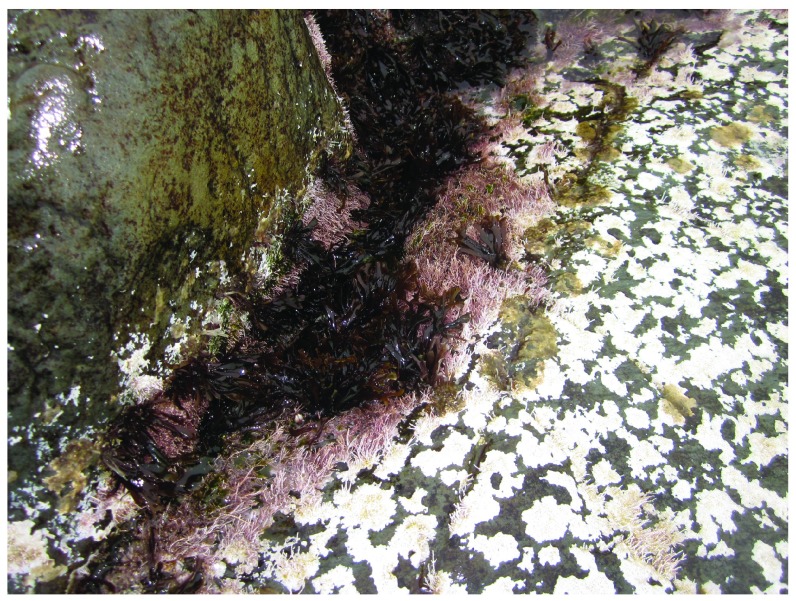
Tor Bay Provincial Park after ice scour. Picture taken at low tide on 27 April 2014 at the wave-exposed site from Tor Bay Provincial Park shown in Figure 6. This picture shows the post-ice survival of some algae in protected sites.

**Figure 10.**
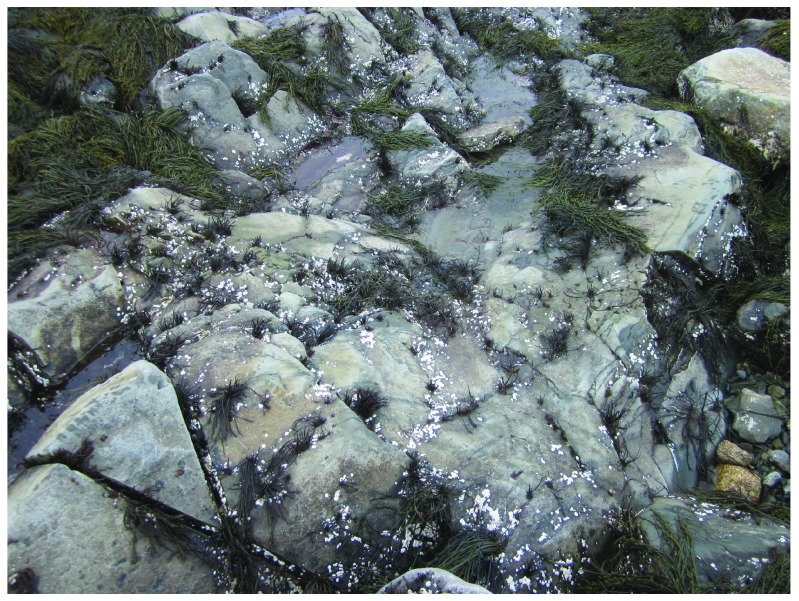
Tor Bay Provincial Park after ice scour. Picture taken at low tide on 27 April 2014 at a wave-sheltered site in Tor Bay Provincial Park, showing the loss of the *Ascophyllum nodosum* canopy that had previously covered these habitats for an undetermined number of years (at least 10, based on observations by R.A.S.). Remains of *A. nodosum* canopies are seen in the upper-left corner and upper-right corner of this picture.

The ice scour that occurred on those shores for days until the ice melted removed a large amount of algae and invertebrates. The duration of the presence of sea ice on the shore was related to the intensity of biological damage. For instance, in Whitehead, which sustained 9 full days (between 4–12 April) of ice coverage ( Canadian Ice Service), the intertidal zone underwent an almost total loss of organisms ( Figure 7). At Tor Bay Provincial Park, which sustained 4 days (between 6–9 April) of ice coverage (likely because it is farther away from the ice source), biomass losses were also high ( Figure 8), but some organisms were able to survive in some protected areas ( Figure 9). The magnitude of ice scour in mainland Nova Scotia in 2014 was such that ice effects were even observed in wave-sheltered habitats. In such habitats, which are normally dominated by the perennial brown seaweed *Ascophyllum nodosum* ^9^, the movement of ice fragments is relatively limited ^5^. However, in 2014, biomass losses were still high in some wave-sheltered habitats, leaving extensive areas without any significant macroalgal coverage ( Figure 10).

## Concluding remarks

**Figure 11.**
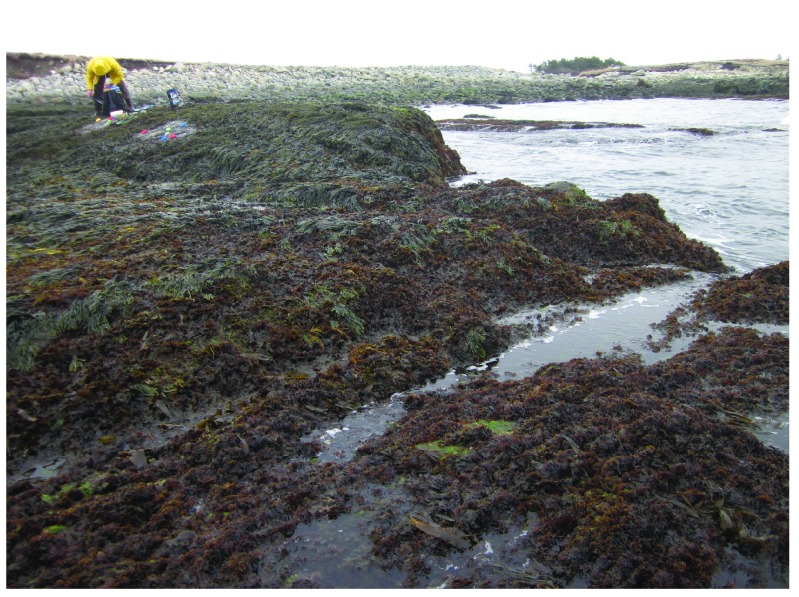
Sober Island after the ice season. Picture taken at low tide on 1 May 2014 at a wave-exposed site in Sober Island, showing a full coverage of seaweed canopies, as the sea ice had not reached this shore during the previous 7 years.

As the duration of the ice presence on the open Atlantic coast of Nova Scotia generally decreases from the Cabot Strait southwards, albeit not linearly ( Canadian Ice Service), the observations herein described suggest that intertidal community structure may be influenced by latitude mediated by ice scour effects. We predict that communities from northern locations in this coastal range would remain in early successional stages, as such places receive drift ice from the Gulf of St. Lawrence mostly every year. Conversely, communities from southern locations in this coastal range might reach more mature stages because of sea ice failing to reach those places for a number of years. This notion is supported by the fact that, on Sober Island (44° 49' 20.3" N, 62° 27' 26.5" W), which is located south of the southernmost reach of the sea ice in 2014 ( Figure 1) and has not been exposed to ice floes since 2007 ( Canadian Ice Service), intertidal communities were well developed and seaweeds extensively covered the rocky surface shortly after the 2014 ice season ( Figure 11). We suggest that a multiannual survey of ice conditions and biological communities along the open Atlantic coast of Nova Scotia could reveal the ecological role that sea ice plays on intertidal community organization in this cold-temperate coastal system.
