# Supplementary material for: Visual record of intertidal disturbance caused by drift ice in the spring on the Atlantic coast of Nova Scotia
Source: F1000Res. 2014 May 16;3:112. [Version 1] doi: 10.12688/f1000research.4146.1 (PMC4118760; doi:10.12688/f1000research.4146.1)
Supplement: Peer review history

# Peer review: Referee response for version 1

**DOI:** 10.5256/f1000research.4439.r4930

**Published:** 2014-05-29

**Authors:** Gregorio Bigatti

**Affiliations:** 1 National Patagonic Center (CENPAT), CONICET, Puerto Madryn, Argentina

This work registered the disturbance caused by drift ice on benthic intertidal communities in rocky shores of Nova Scotia. The authors documented in real time the ice coverage and the latter disturbance of the intertidal algae and invertebrates. It is recommendable to measure the biomass losses if this phenomenon occurs again in the zone. The observations made here are useful to other researchers working in similar habitats, opening new questions on successional stages and the ecological role of sea ice plays on intertidal community organization of benthic communities in Nova Scotia.

I suggest including a map of northern America in Figure 1, and an inset with the location of Nova Scotia and ice drift (actual fig 1).

I have read this submission. I believe that I have an appropriate level of expertise to confirm that it is of an acceptable scientific standard.

# Peer review: Referee response for version 1

**DOI:** 10.5256/f1000research.4439.r4812

**Published:** 2014-05-23

**Authors:** Mathieu Cusson

**Affiliations:** 1 Département des sciences fondamentales, University of Quebec in Chicoutimi, Saguenay, Canada

This observation article provides a portrait of an ice scouring event on the Nova Scotia Atlantic shoreline. The article is very short yet explains and demonstrates well the devastating effects of ice scouring. The authors provide several pictures that efficiently illustrate the disturbed community. It would have been ideal if the authors had taken pictures 4 and 7 as well as 6 and 7 with a common guide mark (for scale) on the ground. However, the pictures are clear enough to appreciate ice scouring impacts on the benthic communities. Such impacts on macrobenthic communities from ice scouring are very common in the St. Lawrence estuary and gulf. I do agree that communities in the latter regions would remain in early successional stages on exposed substrates.

All information provided in this communication is accurate. I consider the observations reported by this paper as interesting and useful.

I would suggest the authors add the reference below into the paragraph 2, first sentence:

Bergeron P, Bourget E (1986) Shore topography and spatial partitioning of crevice refuges by sessile epibenthos in an ice disturbed environment. Mar Ecol Prog Ser 28:129-145

I have read this submission. I believe that I have an appropriate level of expertise to confirm that it is of an acceptable scientific standard.
